# Airborne anaphylaxis: highlighting an invisible enemy

**DOI:** 10.1097/ACI.0000000000000848

**Published:** 2022-08-04

**Authors:** Erminia Ridolo, Cristoforo Incorvaia, Jan Walter Schroeder

**Affiliations:** a Allergy and Clinical Immunology, Medicine and Surgery Department, University of Parma, Parma; b Unit of Allergology and Immunology, ASST Grande Ospedale Metropolitano Niguarda, Milan, Italy

**Keywords:** airborne anaphylaxis, drug allergy, food allergy, inhalation, occupational allergy

## Abstract

**Purpose of review:**

Airborne anaphylaxis is a rare disorder defined by the occurrence of anaphylactic reactions to inhaled allergens, which may arise not only in occupational exposure but also in common settings. Foods are the most common cause of airborne anaphylaxis, even organic mixtures scents. The other important cause is represented by drugs, while in the wide range of other causes, there are often reports on unique cases. This review aims to make an overview about the potential causes of airborne anaphylaxis, by analysing what is described in literature on this topic.

**Recent findings:**

Concerning epidemiology, no data on specific prevalence of airborne allergy in adults are available. To date, only one study evaluated the specific prevalence of airborne allergy with anaphylaxis to foods in children, resulting in 5.9% of reactions due to exposure to aerosolized foods, compared with 78% of reactions caused by food ingestion. In addition to anaphylaxis, airborne-related reactions may also present with symptoms such as rhino-conjunctivitis, wheezing, dyspnoea and asthma.

**Summary:**

A detailed anamnesis facilitates a correct diagnosis, which allows appropriate therapeutic and preventive interventions, but, similarly to rare diseases in general, only specialized doctors are able to implement it. The assumption of the approach used in emergency medicine for other causes of anaphylaxis, that is referring the patient at discharge to an allergist who will teach the basic notions to recognize symptoms and access the appropriate therapy, would allow the patient to avoid situations of serious danger.

## INTRODUCTION

**FIGURE 1:**
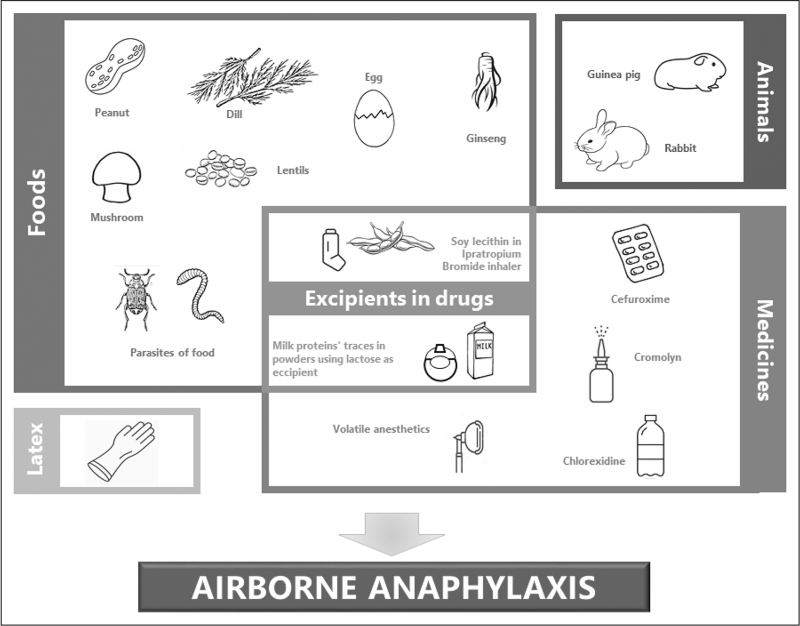
The main causative agents of airborne anaphylaxis.

Through the vision of precision medicine, anaphylaxis is defined as the most severe and life threatening of the allergic reactions, exposing patients to serious risks and requiring rapid diagnosis and management by healthcare providers. In fact, as its symptoms are similar to those of other diseases, for example hives or asthma, recent data suggest that diagnosis is not infrequently wrong [1]. The most common causes of anaphylaxis are drugs, foods and insect stings [2], but further sources are currently considered to be included. Airborne anaphylaxis is defined by the occurrence of anaphylactic reactions to inhaled allergens. It has long been known that such contact via inhalation can cause even severe allergic reactions, which can arise in occupational exposure as well as in common settings including household, school, restaurants and air travel [3]. Although reactions to food through ingestion are triggered by specific proteins [4], these allergens are usually absent in the airborne component: for example, the scent of peanuts, which is contained in smaller organic mixtures, is unable to trigger a common allergic response [5]. As far as epidemiology is concerned, the most recent study reported that the prevalence of food allergy is still increasing, particularly in adults, but no data on specific prevalence of airborne allergy were provided [6]. An epidemiological evaluation of a group of children with anaphylactic reactions to foods with a median age of 7 years reported that most reactions (78%) occurred after ingestion, eight (16%) of them occurred after exclusive skin contact, and three (5.9%) occurred after exposure to aerosolized food [7]. This review aims to make an overview about the potential causes of airborne anaphylaxis (Fig. 1), by analysing and bringing order to what is described in literature on this argument, for the most part consisting of case reports, whose diagnostic tests are summarized in Table 1. 

**Box 1:**
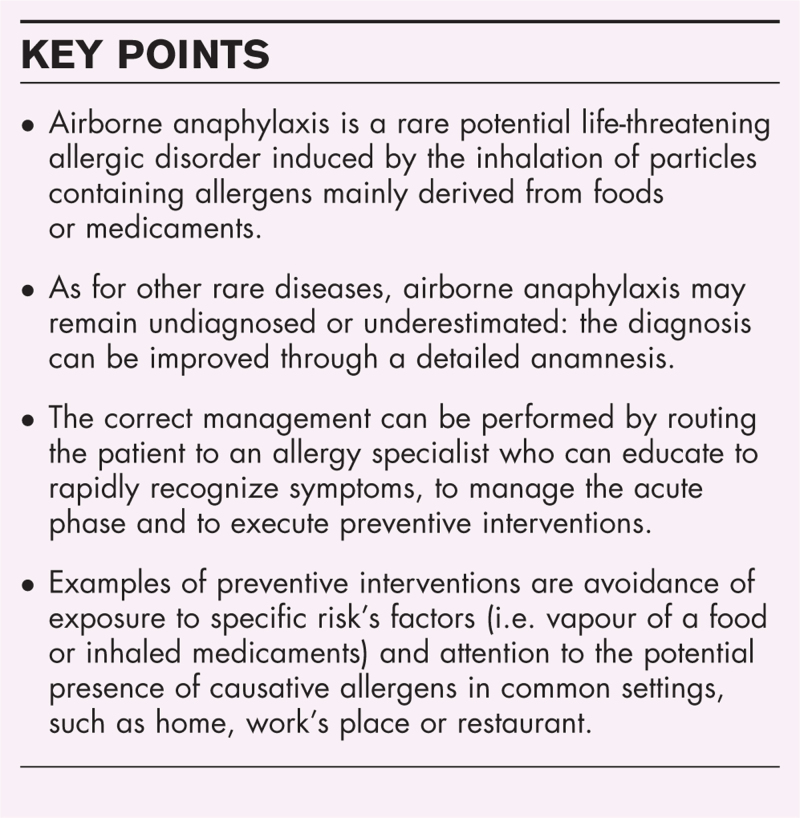
no caption available

**Table 1:** Diagnostic tests performed in most of the case reports of airborne anaphylaxis described

	In-vitro tests				In-vivo tests				
	Total IgE	sIgE	BAT	Baseline tryptase	SPT	Prick-by-prick	ID	Specific inhalation challenge	Other allergies reported
American ginseng powder [14]			+		+				
*Anisakis simplex*[22]	7490 IU/ml	>100 kU/l		6.1 μg/l	+			+	Clam sIgE 5.97 kU/l
*Bruchus pisorium*[21]					+			+	
Cefuroxime [28]	18 kU/l	0.13 kU/l	+		+			+	BAT for cefotaxime: +sIgE and BAT for ceftriaxone: +BAT for cefazolin: +SPT and BAT for ceftazidime: +BAT for ampicillin: +ID for benzylpenicillin 0.01 IU/ml: +
Chlorexidine [24]	38.6 kU/l	2.23 kU/l			+				
Cromolyn sodium [29]	1759 IU/ml				+ in passively sensitised sites^a^				SPTs to bermuda, timothy, bahia, Kentucky blue, orchard, house dust mites, *Cladosporium*, dog and cat dander: +
Dill [20]		- (IgG4: +)			+	-			
Egg-albumin [13]	1879μg/l	+			+				SPTs for egg-yolk and cow's milk: +
Fig (*Ficus carica*) [18]	93.6 kU/l	15.5 kU/l		4.12 mgA/l	+ (fig and fig leaf)	+			SPT for olive tree pollen: +
Guinea pig [33]	200 kU/l	22.7 kU/l							SPTs for dust mites and cat: +sIgE *DermatophagoidesPteronyssinus:* 10.6 kUa/lsIgE *DermatophagoidesFarina*: 11.4 kUa/lsIgE cat dander: 0.89 kUa/l
Latex [31]					+				
Lentil [15]	846 IU/ml	16.7kU/l							sIgE for chickpea: 16.7kU/l
Oyster mushrooms [19]	72IU/ml					+		+	
Rabbit [34]		0.332 kU/l			+				sIgE to guinea pig: 0.031 kU/l
Rice [17]					+			+	SPT for peanut: +
Sevoflurane [27]			+		+		+		

The original units of measurement are reported. BAT, basophil activation test; ID, intradermal test; sIgE, specific IgE; SPT, skin prick test.

a This test was performed in 1981 by passive transfer experiments with serum drawn from the patient 3 months after the reaction and challenged in injection sites with 1% cromolyn solution. The recipient's skin showed no reaction directly to Cromolyn challenge.

## CAUSES OF AIRBORNE ANAPHYLAXIS

### Foods

It is not surprising that one of the most important causes of airborne allergic reactions food-induced is represented by one of the best known causes for anaphylaxis overall. In a large study on 1411 Canadian children with peanut allergy, the annual incidence rate of clinical reaction after accidental exposure to peanuts was 12.5%, with 4.8% of reactions being related to inhalation [8]. Such mechanism was confirmed in an experimental study showing that mice exposed to inhaled peanuts twice a week for 4 weeks developed peanut-specific IgE, IgG1 and IgG2 and showed clinical signs of anaphylaxis [9]. Moreover, the exposure by inhalation to peanut distributed to passengers during air flights has long been responsible for allergic reactions even in passengers who refused it knowing they were allergic. The elimination of the delivery of peanuts by various airline companies proved to be very helpful to reduce this problem. However, in a recent review on the current state of peanut allergy, Abrams *et al.*[10^▪▪^] stated that the risk of fatal childhood anaphylaxis is very low, and that it is minimal considering cutaneous or inhalational exposure.

For what concerns milk's allergy, it is important to take into account that lactose is a common excipient used in the production of many medicaments, likely to be contaminated by milk's proteins. As a proof of this, there is the case of a child who experienced an anaphylactic reaction after the inhalation of Inavir powder (Laninamivir Octanoate Hydrate) as treatment of flu infection. In particular, the drug was examined by western blotting that identified the presence of β-lactoglobulin's traces [11]. Furthermore, respiratory virus infections may contribute their own in the genesis of an inhalation-induced anaphylaxis. In another study, it has been observed how nebulized ovalbumin used as an aeroantigen in normal mice did not take effect, while those infected with respiratory syncytial virus or influenza A virus had a collapsed response without inducing specific serum antibodies. Mice with collapsed response to cutaneous ovalbumin were found to have IgG1 specific to ovalbumin. The authors suggested that infection with respiratory viruses strongly enhances cellular and humoral immune responses to aeroantigen, paving the way for experimental models to investigate such effects [12]. However, studies on humans comparing healthy individuals to patients with respiratory viruses were not performed so far.

It is clear how, in cooking, those that seem to be innocuous powders may turn into a real danger in sensitized individuals, as in the case of two children who experienced both a severe anaphylactic reaction characterized by acute respiratory distress, just being in the same room wherein a pavlova mix (containing egg-albumin) was employed [13]. In a similar way, two paediatric patients (the first a 6-year-old girl and the second a 3-year-old boy) were admitted to the hospital with anaphylaxis and recurrent allergic conjunctivitis, respectively, after exposure to aerosolized powdered American ginseng. The first patient had positive skin prick test to American ginseng, while in the second patient, no IgE-mediated allergic reaction during oral challenge with American ginseng powder was found [14].

As regards reactions potentially caused by legumes, the role of the main protagonist, apart from peanut, is played by lentil. In 2010, a case of anaphylaxis induced by inhalation of airborne lentil particles in cooking fumes was reported [15]. Another case concerned a 22-month-old child with a previous history of angioedema and laryngeal obstruction after the second assumption of lentils in her diet, who exhibited signs of urticaria and anaphylaxis after inhalation of cooked lentils vapours [16].

In the matter of cereals, it is well known that the ingestion of rice is sometimes related to a number of symptoms such as asthma, rhinitis, eczema and gastrointestinal disorders, but not much is known about its potential as an airborne allergen. There is only a report in literature regarding an 8-year-old child who developed a severe anaphylaxis, the proximate cause of which was the inhalation of steam from rice during cooking [17].

In the context of fruit, a severe reaction to figs is described. A 10-year-old boy experienced upper limbs and face itching, eyelid and lip oedema, cough, dyspnoea and dysphagia while striking figs under a fig tree with a tennis racket. There was no correlation with eating, and the reaction trigger was identified in a new protein that has not been described at that time [18].

Sometimes, airborne anaphylaxis can occur as a direct consequence of an occupational allergy. In 2021, the case of a 32-year-old nonatopic farm worker was reported, who experienced respiratory symptoms 30 min after the exposure to oyster mushrooms (*Pleurotus ostreatum*), the spores of which are known as potent allergens. Three days later, in the farm again, she presented dyspnoea, weakness, hives and skin hitching; therefore, after alerting her allergist, she was immediately submitted to a spirometry, that, in comparison to a control spirometry subsequently performed in stable conditions, demonstrated an effective bronchial obstructive answer to mushroom exposure (FEV1 2.13 l and 57% ref. versus FEV1 3.42 l and 91% ref.). A month later, the patient was exposed again to the allergen for packing mushrooms and ten minutes after the exposure she experienced dyspnoea, tachycardia and urticaria with the need of ambulance intervention. Ultimately, after excluding a sensitization to common inhalants and foods through skin prick tests, the allergy to oyster mushroom was confirmed by a prick-to-prick test and a specific inhalation challenge test [19].

Even herbs used to flavour dishes sometimes may act as airborne allergens, as in the case of dill (*Anethum graveolens*), a spice classified in the family of carrot and parsley (*Apiacea*). A 40-year-old woman suffering from seasonal and perennial allergic rhinitis successfully treated with nasal corticosteroids and antihistamines reported that each time she ate foods containing dill, she immediate manifested symptoms including throat tightness and palatal itching, followed by generalized urticaria, vomiting and diarrhoea. Such symptoms progressed with every successive exposure, and, at the time of presentation to physician, they occurred even after inhaling foods cooked with dill [20].

Parasites of food are definitely no exception in the triggering of airborne anaphylaxis. Infestation by parasites species, in fact, may cause, through some of their proteins, hypersensitivity reactions from both skin contact and inhalation. As *Bruchus* species are common habitual parasites of legumes, a study was aimed at investigating in patients with symptoms of immediate hypersensitivity (including contact urticaria, asthma and anaphylaxis) connected to inhalation of the dust of peas infested by *B. pisorum*. The results showed a positive response to prick testing, provocation testing and immune detection to parasitic pea extracts and *B. pisorum*. The authors concluded that the entrance by inhalation or puncture of setae released by *B. pisorum* may be a cause of contact urticaria, anaphylaxis and asthma [21]. As regards nematodes, *Anisakis simplex* infests fishes and cephalopods, causing a number of clinical disorders, including occupational respiratory disease, dermatitis and anaphylaxis. Following a study of allergic airborne asthma caused by *Anisakis simplex*[22], the case of a nonatopic woman was described. After having urticaria and angioedema from eating fresh anchovy, she developed rhino-conjunctivitis, pruritus, tongue oedema, cough and dyspnoea just by standing on the street, in front of a fish store. The results of diagnostic procedures proved the responsibility of *Anisakis* through airborne contact [23].

### Medications

Drugs may be responsible for many severe allergic reactions and even the inhalation of medications, intended as active substance or any of their excipients, may trigger episodes of anaphylaxis.

Chlorhexidine is a commonly used antiseptic and disinfectant (also present in mouthwash, ointment, toothpaste and nose and eye-drops), which can cause both immediate and delayed allergic reactions, including anaphylaxis. Three cases of reactions from occupational exposure were reported in 2013, with confirmation from placebo-controlled specific challenge tests by inhalation. One patient had a systemic reaction to environment exposure [24]. Five years later, a case of severe anaphylaxis occurring in the workplace was described, highlighting the importance for clinicians of being aware of possible risks of severe allergic reactions to chlorhexidine in the occupational field [25].

Several reactions to anaesthetic drugs are described. In 2011, a 54-year-old man with a previous history of an inhalation lung injury from butane gas fuel had been hospitalized because of breath shortness. When receiving Lidocaine aerosol to prepare for bronchoscopy, the patient had an asthmatic attack and an anaphylactic shock with respiratory arrest requiring cardiopulmonary resuscitation followed by admission to the ICU and intubation for 3 days. The authors suggested that aerosolized Lidocaine anaesthesia may induce severe condition as airway narrowing and anaphylactic shock, and that practitioners should be mindful of this potential severe complication [26]. Moreover, in a 6-year-old child undergoing adenotonsillectomy, who had an anaphylactic reaction which required testing a number of the drugs used to detect the trigger, only the volatile anaesthetic sevoflurane gave a positive result. Considering the recentness of the observation, the authors claimed that also volatile anaesthetics can be responsible of anaphylactic reactions and therefore should be included in the list of anaesthetics to be tested in case of severe reactions during anaesthesia [27]. The theme of the airborne anaphylaxis in occupational allergy occurs even for the medicaments. For example, for a 53-year-old nonatopic nurse with recurrent anaphylactic reactions at work, after excluding responsibility of drug-specific tasks, the cause remained unidentified until another severe anaphylaxis arose after oral use of Cefuroxime to treat a respiratory infection. The causative role of Cefuroxime was confirmed by specific IgE, skin prick test and basophil activation test. The occurrence of generalized urticaria after a cumulative dose of about 10 μg of the drug by inhalation challenge confirmed the diagnosis. After 1 year of complete stopping of exposure, no further allergic reactions were observed [28].

If it were not a life-threatening question, it would be ironic notice that even medicines employed to treat respiratory issues may be included in the causes of airborne severe allergic reactions. The first report of anaphylaxis to Disodium Cromoglycate (Cromolyn) dates back to 1981, when a 7-year-old girl suffering from asthma attacks occurring one to two times a year, was prescribed Cromolyn as added treatment because of increasing frequency of asthma episodes. Its first inhalation caused immediate cyanosis and thready weak pulse, which required resuscitation measures. On arrival at hospital, the patient received Epinephrine, Sodium Bicarbonate, intravenous Isoproterenol and Dexamethasone, along with mechanical ventilation. The absence of IgG anti-Cromolyn antibody suggested that the events following the administration of Cromolyn were likely to have accounted for IgE stimulation of other antibody classes [29]. Sometimes, the recognition of the basic cause of anaphylaxis induced by inhalation may be even more difficult because of the presence of hidden elements. A 44-year-old woman with a history of airway disease and allergy to peanut and soy had an anaphylactic reaction after inadvertent ingestion of peanut oil. After a successful treatment with Epinephrine, corticosteroids and antihistamines, at 3 and 6 h after admission, the patient developed recurrent airway obstruction, hypotension and generalized skin eruption while receiving inhalation therapy with Ipratropium Bromide by metered-dose inhaler. Careful research revealed that the drug contained soy lecithin as an inert component, and discontinuing its use resulted in whole resolution of the symptoms. The authors highlighted that physician treating patients allergic to soy should be aware of the potential risk of severe reactions to Ipratropium Bromide therapy [30].

### Further causes

Latex is a well known cause of severe reactions in hospital settings that, despite the efforts to lessen it through by reducing its use and extending latex-free medical devices, continues to be a health problem in many countries [31]. It should be kept in mind that airborne latex particles can also cause severe reactions. In a woman in her first pregnancy, the exposure to airborne latex particles caused an anaphylactic reaction, which resulted in foetal distress with nearly need of an emergency caesarean delivery and in the need for support of maternal circulation with intravenous fluids and administration of vasopressors and oxygen. The rapid resolution of symptoms after latex source removal avoided more aggressive interventions [32].

Unfortunately, also beloved pets can occasionally hide traps. A patient who had kept a guinea pig for 2 years had anaphylaxis after close contact with such pet, while other pets such as cats and dogs did not elicit any allergic symptoms. Allergen challenge testing was not performed to prevent possible anaphylactic reactions and the ultimate diagnosis has been based on contact history, clinical characteristics and allergen test results [33]. In another incident, rabbit has been involved. A patient in paediatric age with a history of seasonal allergic rhinitis and eczema had an episode of dry cough soon after incoming a place where there was a live rabbit in a cage, with no direct contact. After leaving the place, cough disappeared spontaneously. Entering the same place after some weeks, the new exposure to the rabbit in the cage resulted within minutes in cough, wheezing, rhinorrhoea, nasal and ocular pruritis, sneezing paroxysms, shortness of breath and feeling of throat swelling. Even after letting the place, symptoms proceeded, also adding severe hoarseness, difficulty breathing and croup-like cough. After quite a lot of years, the patient, having had no more contact with rabbits, tolerated other pets such as cats and hamsters and denied symptoms anywhere else but home [34].

## ASSESSMENT OF THE FEW AVAILABLE REVIEWS IN LITERATURE

The first observation of a variant of anaphylaxis induced by inhalation was reported in 1982 in patients undergoing inhaled allergen challenge who developed anaphylactic reactions. The authors suggested that inhaled allergens could be a frequent cause of repeated anaphylaxis when other known causes of anaphylaxis are excluded [35]. Today, we know that airborne anaphylaxis is much less frequent than that induced by common causative agents, but it is still dependent on different triggers. A limited number of review articles are available, but only those dedicated to foods have evaluated a sufficient number of patients to draw reliable considerations. James and Crespo [36] evaluated the articles published up to 2007 on food allergic reactions caused by inhalation exposure to airborne food allergens. The range of symptoms included reactions such as rhino-conjunctivitis, wheezing, dyspnoea, asthma and anaphylaxis. The authors suggested that the evaluation of food allergy should be considered not only in patients presenting with suspected food allergy through ingestion but also in those presenting with possible exposure to aerosolized food particles through inhalation. The medical history supplemented with appropriate laboratory testing and food challenges can afford useful information in the workup of patients with suspected airborne food allergens [36]. The most extensive review on hypersensitivity reactions caused by inhalation was done by Leonardi *et al.* [37], who considered it a problem of increasing importance in children. A number of foods were found to trigger reactions by inhalation, including cow's milk, peanut, bean, buckwheat, poppy seed, chickpeat, potato, rice, fish, sesame, lentil and lupine. The authors warranted further studies to define the accuracy of diagnostic tests and the prevalence, incidence and natural history of food allergy through inhalation route [37].

## CONCLUSION

Current knowledge on airborne anaphylaxis defines it as a rare allergic disorder, which, however, can be associated with even serious, potentially life-threatening reactions. A large number of agents can trigger airborne allergy and in particular anaphylaxis, but foods and medications have the greatest importance in terms of both frequency and severity of reactions. It is necessary reminding, in fact, that even if food is conceived as a major responsible factor for anaphylaxis induced by inhalation, sometimes the effective cause can reside elsewhere, as in the case of particular inhaled medicines or in common settings such as home or work's place. As it often happens with rare diseases, airborne allergy may also go undiagnosed, exposing the patient to the risk of reactions of increasing severity. So, once again, a specific anamnesis shows itself as a crucial instrument for a faster and correct diagnosis. An improvement in the diagnosis and in the subsequent patient's management may be obtained with the approach used in emergency medicine in patients with anaphylactic reactions to insect stings, referring the patient to an expert on this allergy upon discharge. The evaluation of a specialist who can educate the patient on the risk factors for airborne allergy anaphylaxis would allow the avoidance of situations of serious danger, such as exposure to vapours of the food cooked to which the patient is allergic, especially if the vapour concentration is high and the duration of exposure is prolonged, as it can occur in restaurants.

## Acknowledgements

*None.*

### Financial support and sponsorship

*None.*

### Conflicts of interest

*There are no conflicts of interest.*

## REFERENCES AND RECOMMENDED READING

Papers of particular interest, published within the annual period of review, have been highlighted as:

▪ of special interest
▪▪ of outstanding interest
